# GUCY2C signaling limits dopaminergic neuron vulnerability to toxic insults

**DOI:** 10.21203/rs.3.rs-3416338/v1

**Published:** 2023-10-13

**Authors:** Lara Cheslow, Matthew Byrne, Jessica S. Kopenhaver, Lorraine Iacovitti, Richard J. Smeyne, Adam E. Snook, Scott A. Waldman

**Affiliations:** 1 Department of Pharmacology, Physiology, & Cancer Biology, Thomas Jefferson University, Philadelphia, PA, USA; 2 Department of Neurosciences, Thomas Jefferson University, Philadelphia, PA, USA; 3 Department of Microbiology & Immunology, Thomas Jefferson University, Philadelphia, PA, USA; 4 Sidney Kimmel Cancer Center, Thomas Jefferson University, Philadelphia, PA, USA

**Keywords:** GUCY2C, dopaminergic neurons, cyclic GMP, MPTP, mitochondria, ROS, Parkinson’s disease

## Abstract

Mitochondrial dysfunction and reactive oxygen species (ROS) accumulation within the substantia nigra pars compacta (SNpc) are central drivers of dopaminergic (DA) neuron death in Parkinson’s disease (PD). Guanylyl cyclases, and their second messengers cyclic (c)GMP, support mitochondrial function, protecting against ROS and promoting cell survival in a number of tissues. However, the role of the guanylyl cyclase-cGMP axis in defining the vulnerability of DA neurons in the SNpc in PD remains unclear, in part due to the challenge of manipulating cGMP levels selectively in midbrain DA neurons. In that context, guanylyl cyclase C (GUCY2C), a receptor primarily expressed by intestinal epithelial cells, was discovered recently in midbrain DA neurons. Here, we demonstrate that GUCY2C promotes mitochondrial function, reducing oxidative stress and protecting DA neurons from degeneration in the 1-methyl-4-phenyl-1,2,3,6-tetrahydropyridine (MPTP) mouse model of neurodegeneration. GUCY2C is overexpressed in the SNpc in PD patients and in mice treated with MPTP, possibly reflecting a protective response to oxidative stress. Moreover, cGMP signaling protects against oxidative stress, mitochondrial impairment, and cell death in cultured DA neurons. These observations reveal a previously unexpected role for the GUCY2C-cGMP signaling axis in controlling mitochondrial dysfunction and toxicity in nigral DA neurons, highlighting the therapeutic potential of targeting DA neuron GUCY2C to prevent neurodegeneration in PD.

## Introduction

Parkinson’s disease (PD) is the second most common neurodegenerative disorder in the US, affecting ≥ 1% of adults over 60 years of age.^1, 2^ Motor deficits including resting tremor, cogwheel rigidity, and bradykinesia, reflect selective loss of dopaminergic (DA) neurons within the substantia nigra pars compacta (SNpc) and subsequent depletion DA in the dorsal striatum.^1–3^ Current PD treatments mitigate clinical symptoms by raising striatal DA levels (such as levodopa (L-DOPA), monoamine oxidase (MAO) inhibitors, and catecholamine-O-methyl-transferase inhibitors) or mimicking the effects of DA signaling in the striatum (such as DA agonists)^2–4^ but do not slow DA neurodegeneration and disease progression. Beyond the inability of PD medications to intervene in SNpc degeneration, there are no FDA-approved predictive biomarkers for detecting PD progression prior to SNpc neurodegeneration and development of clinical symptoms.^5^ Given the burden of disease, there is an urgent need to develop novel approaches for early detection and effective treatment of PD.

The etiology of PD is complex and multifaceted. Of PD cases, 10–15% are familial, and >20 gene mutations have been linked to PD development, including glucocerebrosidase (GBA), alpha-synuclein (SNCA), leucine rich repeat kinase 2 (LRRK2), PTEN-induced kinase 1 (PINK1), parkin, and parkinsonism-associated deglycase (DJ-1, also known as PARK8).^1, 6–12^ However, most PD cases are sporadic and have been closely correlated with environmental factors, including exposure to heavy metals, agricultural pesticides, and influenza infection.^13–22^ A mechanistic link uniting the broad range of risk factors for PD development was discovered in 1982, when patients who inadvertently injected contaminated heroin presented to clinic with L-DOPA-responsive PD-like symptoms. These patients had unknowingly consumed 1-methyl-4-phenyl-1,2,3,6-tetrahydropyridine (MPTP), a lipophilic compound that crosses the blood-brain barrier (BBB) and is metabolized by MAO-B to its active form, 1-methyl-4-phenylpyridinium (MPP+).^23^ MPP+ is selectively taken up by DA transporter (DAT) expressed by DA neurons, and inhibits complexes I and IV of the mitochondrial electron transport chain (ETC), leading to an accumulation of reactive oxygen species (ROS) and apoptosis.^24–26^ The discovery of MPTP as a causative agent of Parkinsonism introduced impaired mitochondria as a crucial driver of pathology. Despite the wide range of factors contributing to PD, impaired mitochondrial respiration and turnover and elevated levels of ROS within SNpc DA neurons are nearly ubiquitous among PD patients.^27–31^

Guanylyl cyclase C (GUCY2C) is a transmembrane receptor expressed in the intestinal epithelium, hypothalamus, ventral tegmental area (VTA), and nigrostriatal pathway.^32–35^ Upon stimulation with exogenous ligands heat-stable enterotoxin (STa) or linaclotide (LIN), or endogenous intestinal hormones guanylin and uroguanylin, GUCY2C catalyzes the conversion of guanosine triphosphate (GTP) to cyclic guanosine monophosphate (cGMP).^36, 37^ Mice from which GUCY2C has been eliminated [GUCY2C Knockout (KO), *Gucy2c*^−/−^] mice have dysregulated mitochondria, elevated ROS, and oxidative damage in gut epithelia,^38^ suggesting a potential parallel role for the receptor in other cells expressing GUCY2C beyond the intestine.

The present studies demonstrate that GUCY2C is expressed within midbrain DA neurons, and loss of GUCY2C increases ROS and reduces mitochondrial function in the nigrostriatal pathway. Indeed, the absence of GUCY2C creates a vulnerability in SNpc, with DA neuron loss and gliosis following subtoxic doses of MPTP, suggesting that GUCY2C plays a protective homeostatic role in DA neurons. In that context, GUCY2C is increased in the SNpc of patients with PD and mice exposed to MPTP, suggesting a protective feedback loop between dysfunctional mitochondria associated with oxidative stress and GUCY2C-cGMP signaling. Indeed, cGMP enhances antioxidant capacity and cell survival, protecting against MPP+-induced ROS upregulation, loss of mitochondrial integrity, and cell death in DA neurons *in vitro*.

These observations reveal an unexpected role for the GUCY2C-cGMP signaling axis in regulating the toxic vulnerability of nigral DA neurons. Indeed, GUCY2C may be a therapeutic target for early intervention to protect against DA neuron degeneration. As GUCY2C is specifically expressed on DA and hypothalamic neurons within the CNS,^35^ targeting GUCY2C to raise cGMP within DA neurons will have limited off-target effects. We also reveal a novel potential feedback loop, identifying GUCY2C as a transcriptionally regulated target in response to oxidative stress in DA neurons. In addition to the therapeutic potential of targeting GUCY2C, this discovery suggests that GUCY2C may hold potential as a biomarker for early PD pathogenesis prior to significant DA neuron death.

## Results

### Functional GUCY2C is expressed by DA neurons within the SNpc.

**Figure 1.**
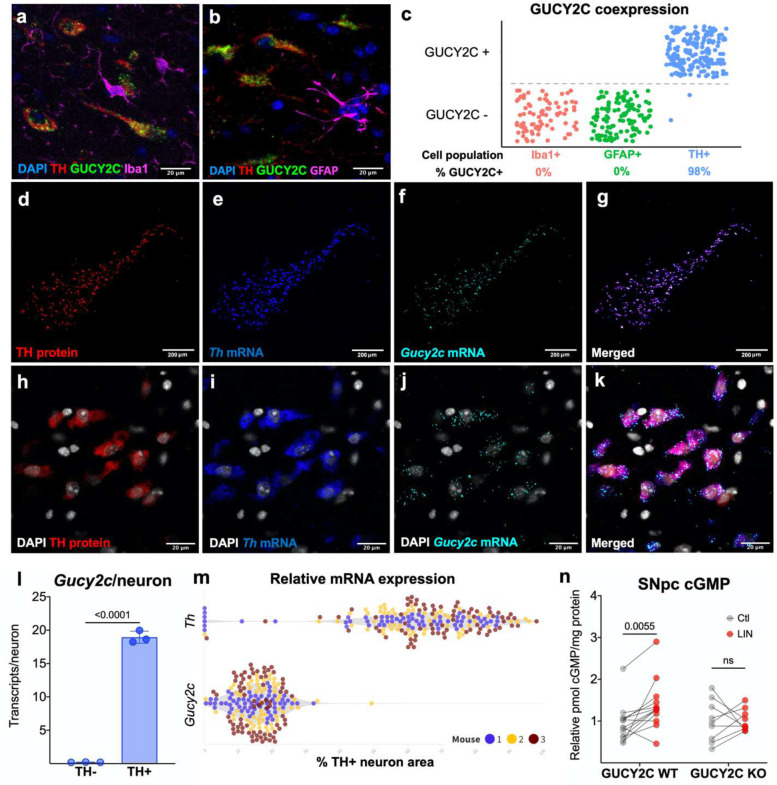
Functional GUCY2C protein and mRNA are expressed by DA neurons within the SNpc. **(a-c)** Immunofluorescent staining reveals that GUCY2C protein is expressed in 98% of TH+ neurons, but not in astrocytes or microglia, in the mouse midbrain. (d-k) Combined immunofluorescence and RNAscope identifies high levels of *Gucy2c* mRNA co-expressed with TH protein and mRNA. (l) *Gucy2c* mRNA is not expressed by TH-negative cells. (m) *Gucy2c* mRNA is expressed at nearly a third of *Th* mRNA levels in DA neurons. (n) Treating *Gucy2c*^+/+^, but not *Gucy2c*^−/−^, SNpc with the GUCY2C agonist (LIN) upregulates intracellular cGMP production. Statistics calculated using 2-way ANOVA with false discovery rate <0.05.

Previously, we demonstrated that GUCY2C is transcribed and translated in the SNpc prior to being trafficked to the striatum.^35^ GUCY2C protein (Fig. 1a–c) and mRNA (Fig. 1d–l) are expressed on 98% of TH+ neurons in the SNpc, but not on microglia or astrocytes. GUCY2C mRNA is expressed at nearly a third of TH mRNA levels in DA neurons (Fig. 1m). The GUCY2C agonist LIN stimulates production of intracellular (Fig. 1n), but not extracellular (supplemental Fig. 1), cGMP in SNpc from wild type (WT; *Gucy2c*^+/+^), but not GUCY2C KO (*Gucy2c*^−/−^), mice demonstrating the utility of targeting SNpc GUCY2C to raise intracellular cGMP levels specifically in DA neurons.

### Loss of GUCY2C leads to mitochondrial dysfunction and oxidative stress within the nigrostriatal pathway.

**Figure 2.**
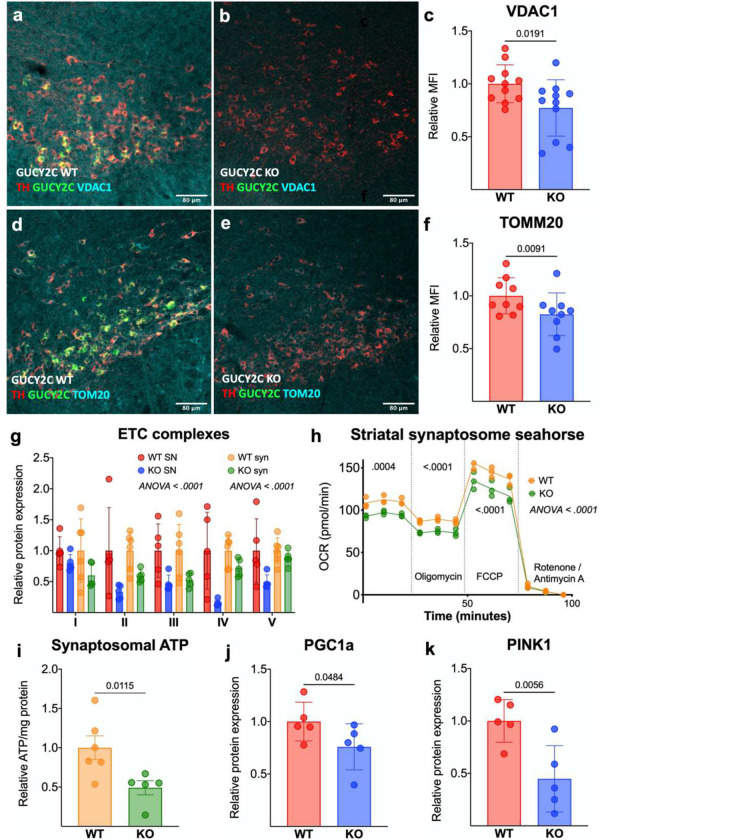

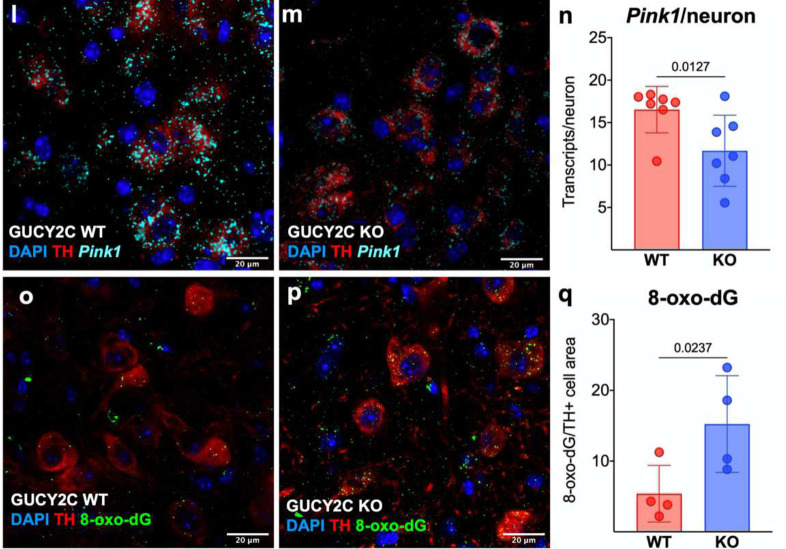
Loss of GUCY2C leads to mitochondrial dysfunction and oxidative stress within the nigrostriatal pathway. (a-f) Immunofluorescence of mouse midbrain reveals lower levels of (a-c) VDAC1 and (d-f) TOMM20 in *Gucy2c*^−/−^ SNpc (n=9–11). (g) Protein isolated from TH-GFP *Gucy2c*^+/+^ and *Gucy2c*^−/−^ SNpc and striatal synaptosomes reveals a lower level of ETC complex proteins in the *Gucy2c*^−/−^ nigrostriatal pathway (n=5). (h) Seahorse analysis reveals lower rates of oxygen consumption in *Gucy2c*^−/−^ striatal synaptosomes (n=3). (i) *Gucy2c*^−/−^ striatal synaptosomes produce lower levels of ATP at baseline (n=5–6). (j-k) *Gucy2c*^−/−^ mice express lower levels of PGC1a and PINK1 protein in the SNpc, quantified by immunoblot (n=5). (l-n) *Gucy2c*^−/−^ mice express fewer *Pink1* mRNA transcripts within DA neurons quantified by RNAscope (n=7). (o-q) Immunofluorescence of 8-hydroxy-2-deoxyguanosine (8-oxo-dG) in *Gucy2c*^+/+^ and *Gucy2c*^−/−^ SNpc reveals greater levels of mitochondrial DNA (mtDNA) oxidation in GUCY2C^−/−^ (n=4). Statistics calculated using 2-way ANOVA with false discovery rate (g-h) or one-tailed t-test (c, f, i, j, k, n, q). All immunoblot analyses were performed using microdissected TH-GFP reporter tissue.

Previously, we demonstrated that GUCY2C promotes mitochondrial integrity in the gut by maintaining mitochondrial protein content and oxygen consumption in intestinal epithelium.^39^ In that context, loss of GUCY2C in the SNpc leads to lower levels of outer mitochondrial proteins VDAC1 and TOMM20^40^ (Fig. 2a–f). Similarly, SNpcs isolated from GUCY2C KO mice expressing a TH-GFP reporter express lower levels of mitochondrial ETC complexes compared to those from WT mice expressing the reporter (Fig. 2g, supplemental Fig. 2a). SNpc DA neurons project to the dorsolateral striatum,^41, 42^ where DA is released from mitochondria-rich synapses.^43, 44^ Synaptosomes isolated from this region (supplemental Fig. 2b–c) in GUCY2C KO mice express lower levels of ETC proteins (Fig. 2g, supplemental 2d–f), have a reduced oxygen consumption rate (Fig. 2h), and produce lower levels of adenosine triphosphate (ATP) (Fig. 2i). In the context of reduced mitochondrial structure and function, proteins PGC1a, a cGMP-responsive transcription factor that promotes mitochondrial biogenesis,^45^ and the ubiquitin-kinase PINK1, which mediates mitophagy,^46^ are reduced in *Gucy2c*^−/−^ SNpcs (Fig. 2j–k), (supplemental Fig. 2g). Further, *Pink1* mRNA is trafficked within neurons to support local mitophagy, and *Pink1* transcripts have exceptionally short half-lives when unbound from damaged mitochondria.^47–49^ This tightly controlled post-transcriptional regulation offers *Pink1* mRNA analysis as an additional readout of mitochondrial dynamics. In alignment with our protein data, *Pink1* mRNA transcript levels are significantly reduced in individual *Gucy2c*^−/−^ DA neurons (Fig. 2l–n). These findings are especially interesting, given that autosomal recessive mutations in the *Pink1* gene predisposes individuals to hereditary PD.^50^ Further, GUCY2C KO mice have more oxidized mitochondrial DNA (mtDNA) within DA neurons, indicative of a higher ROS burden (Fig. 2o–q).^51, 52^

### GUCY2C KO mice have an enhanced vulnerability to MPTP.

**Figure 3.**
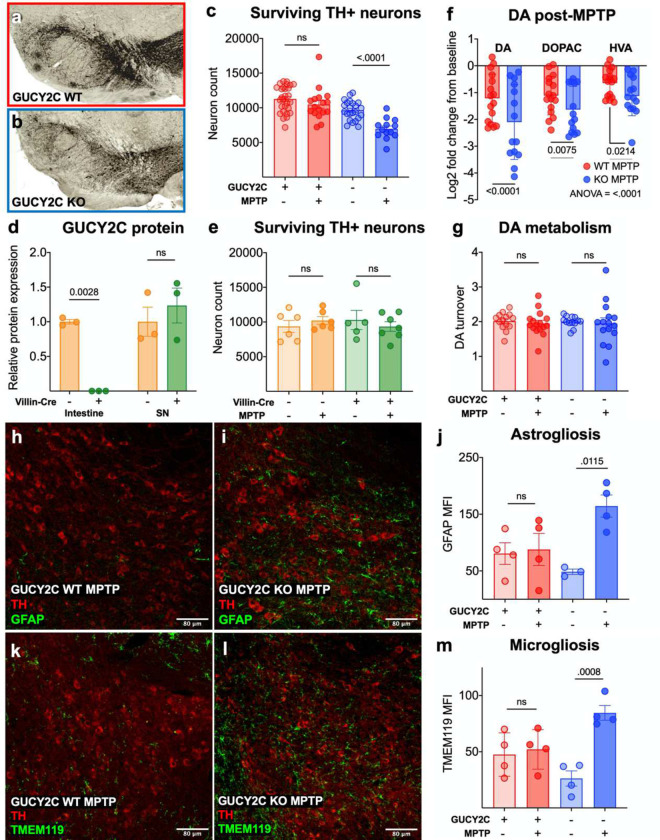
*Gucy2c*^−/−^ mice have an enhanced vulnerability to mitochondrial toxin MPTP. (a-c) Counting surviving TH+ neurons in (a-c) IHC-stained SNpc reveal that *Gucy2c*^−/−^ mice lose more DA neurons post-MPTP (n=13–28). (d-e) Neuron loss is negligible post-MPTP in both Villin^cre+^ x *gucy2c*^fl/fl^ and Villin^cre−^ x *gucy2c*^fl/fl^ mice, indicating that loss of intestinal GUCY2C (d) does not increase vulnerability to MPTP-induced DA neurodegeneration (e) (n=3 for Vil-Cre x *gucy2cf/f/* validation (d); n=5–7 for MPTP experiment (e). (f-g) HPLC reveals that *Gucy2c*^−/−^ mice lose significantly higher levels of DA, 3,4-dihydroxyphenylacetic acid (DOPAC), and homovanillic acid with no impact on DA metabolism. (HVA) post MPTP (n=13–16). h-m. Immunofluorescent analysis of MPTP SNpc demonstrates that levels of (h-j) astrogliosis (GFAP) and (k-m) microgliosis (TMEM119) are significantly greater in *Gucy2c*^−/−^ mice (n=3–4). Statistics were calculated using 2-way ANOVA with a false discovery rate <0.05.

As mitochondrial dysfunction is a major driver of PD, we hypothesized that *Gucy2c*^−/−^ mice with mitochondrial deficits should be uniquely vulnerable to neurodegeneration induced by MPTP, a mitochondrial toxin that selectively targets DA neurons and induces cell death through oxidative stress.^24, 25, 53, 54^ Subtoxic doses of MPTP produce loss of SNpc DA neurons in *Gucy2c*^−/−^, but not *Gucy2c*^+/+^, mice (Fig. 3a–c). This neurodegeneration is limited to the SNpc; subtoxic MPTP does not induce neurodegeneration in the *Gucy2c*^+/+^ or *Gucy2c*^−/−^ VTA (supplemental Fig. 3a–e). Importantly, conditionally eliminating GUCY2C only in intestine, but not brain, (Fig. 3d, Supplemental 3f) did not amplify DA neuron vulnerability to subtoxic doses of MPTP, highlighting the role of SNpc GUCY2C in neuroprotection (Fig. 3e). Beyond loss of DA neurons, subtoxic MPTP induces greater loss of striatal DA and its metabolites in *Gucy2c*^−/−^, as compared to in *Gucy2c*^+/+^, mice (Fig. 3f). Interestingly, *Gucy2c*^−/−^ mice have elevated levels of DA and DOPAC, but not HVA, at baseline compared to *Gucy2c*^+/+^ mice (supplemental Fig. 3g–i), and DA metabolism is comparable between genotypes before and after exposure to MPTP (Fig. 3g). Further, subtoxic MPTP induces reactive astrogliosis and microgliosis in SNpcs from *Gucy2c*^−/−^, but not *Gucy2c*^+/+^, mice (Fig. 3h–m). Increased toxicity of MPTP in *Gucy2c*^−/−^, compared to *Gucy2c*^+/+^, mice does not reflect a downregulation of VMAT2 or overexpression of DAT within DA neurons (supplemental Fig. 3j), or an increase in the expression or protein of MAO-B, the enzyme which converts MPTP to the proximal toxin MPP+ (supplemental Fig. 3k–m).^23, 55, 56^ Further, the differential neurodegeneration between genotypes is not due to a more rapid response in *Gucy2c*^−/−^ mice; subtoxic MPTP does not induce DA neurodegeneration at either 7 or 14 days post-injection in *Gucy2c*^+/+^ mice (supplemental Fig. 3n).

### GUCY2C is overexpressed in pathology.

**Figure 4.**
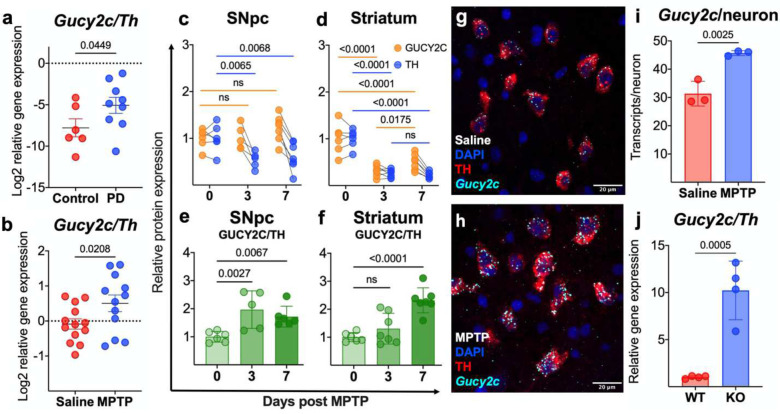
GUCY2C is overexpressed in pathology. ***(a)** Gucy2c/Th* mRNA is elevated in PD patients quantified from microarray analyses (n=6–9) and **(b)** MPTP mice, quantified by qPCR (n=12–13). Immunoblot analysis **(c-f)** reveals that TH is differentially reduced, compared to GUCY2C, in the nigrostriatal pathway following MPTP exposure **(c-d)**, associated with an **(e-f)** elevated GUCY2C/TH ratio in the SNpc and striatum (n=5–7). **(g-i)** RNAscope and immunofluorescence of the SNpc of mice treated with **(g)** saline or **(h)** MPTP reveals **(i)** elevated *Gucy2c* mRNA transcripts in TH+DA neurons following MPTP exposure (n=4–5). **(j)** *Gucy2c*^−/−^ (KO) mice upregulate *Gucy2c* transcription, compared to *Gucy2c*^+/+^ (WT) mice, quantified by qPCR. Statistics were calculated using a one-tailed t-test (a, b, i, j), a two-way AVOVA with a post-hoc false discovery rate <0.05 (c-d), and a one-way ANOVA with a post-hoc false discovery rate <0.05 (e, f).

Publicly available transcriptomic data reveal that *Gucy2c* mRNA is overexpressed in DA neurons in PD patients (Fig. 4a, supplemental Fig. 4a) (GEO GSE42966). Similarly, *Gucy2c*^+/+^ mice treated with toxic doses of MPTP overexpress GUCY2C mRNA and protein in the nigrostriatal pathway (Fig. 4b–f, supplemental Fig. 4b). Notably, while GUCY2C/TH mRNA and protein levels significantly increase in the SNpc three days post-MPTP, increases in striatal GUCY2C/TH protein levels are only observable seven days post-injection. This delay likely reflects the transit time of trafficking translated GUCY2C protein from the SNpc to the striatum.^35^ MPTP-induced upregulation in *Gucy2c* mRNA is caused by a compensatory increase of *Gucy2c* transcription in surviving DA neurons, rather than a selective survival advantage of neurons over-expressing *Gucy2c* (Fig. 4g–i). In contrast to the SNpc, *Gucy2c* mRNA expression is unchanged in the VTA post-MPTP injection, consistent with the relative resilience of the VTA to DA neurodegeneration (supplemental Fig. 4c).^57–64^ Interestingly, overexpression of *Gucy2c* in PD patients and mice treated with MPTP mirrors the expression of mRNA in SNpcs from *Gucy2c*^−/−^ mice (Fig. 4j) which exhibit baseline oxidative stress (Fig. 2o–q). *Gucy2c*^−/−^ mice were generated by inserting a neomycin cassette into exon 1 of the *Gucy2c* gene, allowing expression of *Gucy2c* mRNA, but not protein.^65^ In turn, *Gucy2c* mRNA overexpression is associated with chronic basal oxidative stress and damage in the SNpc in *Gucy2c*^−/−^ mice (Fig. 2o–q).

### Cyclic GMP promotes antioxidant capacity *in vitro* and protects DA neurons from oxidative stress.

**Figure 5.**
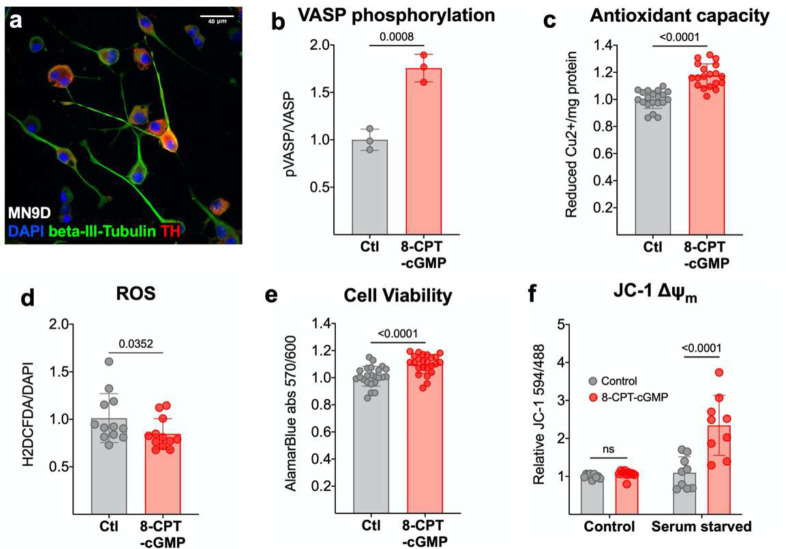
Cyclic GMP promotes antioxidant capacity *in vitro* and protects DA neurons from oxidative stress. **(a)** MN9D neurons accumulate **(b)** pVASP^ser239^ in response to 8-pCPT-cGMP (n=3) quantified by immunoblot analysis. 8-pCPT-cGMP increases **(c)** antioxidant capacity (n=24), which correlates with **(d)** reduced cellular ROS in 8-pCPT-cGMP treated cultures, quantified by CM-H2DCFDA staining (n=12). **(e)** 8-pCPT-cGMP increases cell viability quantified by alamarBlue^™^ reduction (n=24). **(f)** Serum starvation reveals that 8-pCPT-cGMP mitigates loss of mitochondrial membrane potential (ΔΨ_m_) in culture conditions that elevate oxidative stress (n=9). Statistics were calculated using a one-tailed t-test (b-e) and a two-way ANOVA with a post-hoc false discovery rate <0.05 (f).

The protective role of cGMP, the product of GUCY2C signaling, was explored in MN9D mouse DA neurons in vitro.^66^ Treating MN9D neurons (Fig. 5a) with the cell-permeable cGMP analog 8-pCPT-cGMP (they do not express GUCY2C) induces phosphorylation of VASP^ser239^ (Fig. 5b, supplemental Fig. 5), confirming the capacity of these cells to respond to cGMP.^67^ 8-pCPT-cGMP increases the antioxidant capacity (Fig. 5c), reduces cellular ROS (Fig. 5d), and increases viability (Fig. 5e–f) of MN9D neurons. Protective effects of cGMP signaling are further pronounced upon exposure to oxidative stress. Under standard culture conditions, 8-pCPT-cGMP treatment of MN9D neurons has no impact on relative mitochondrial membrane potential (ΔΨ_m_; Fig. 5f), a marker of mitochondrial health.^68^ However, 8-pCPT-cGMP significantly increases ΔΨ_m_ in neurons cultured in serum-starved media a condition that induces oxidative stress.^69–71^

### Cyclic GMP reduces MPP+-induced oxidative stress, mitochondrial dysfunction, and cell death.

**Figure 6.**
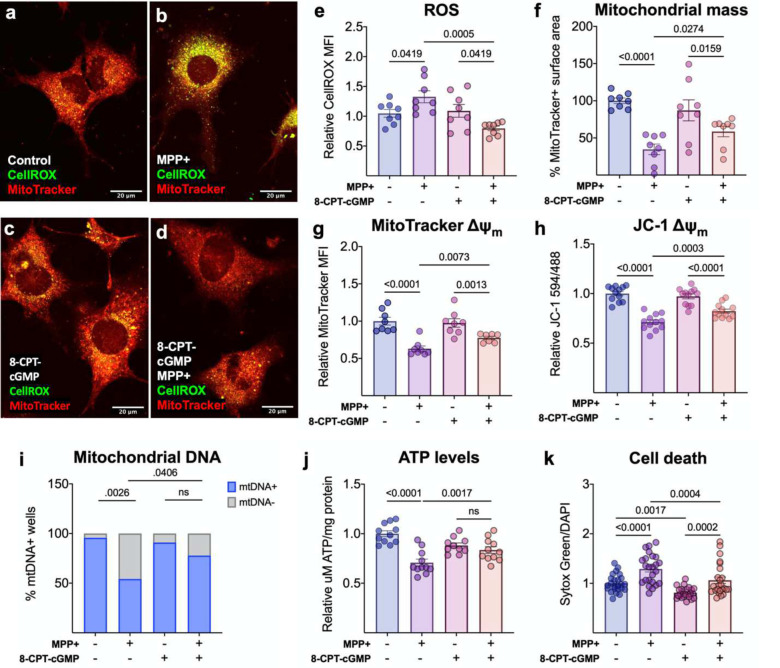
Cyclic GMP reduces MPP+-induced oxidative stress, mitochondrial dysfunction, and cell death. 8-pCPT-cGMP pretreatment rescues MN9Ds from MPP+-induced (a-e) ROS accumulation (n=20–24) and mitigates MPP+-induced loss of (f) mitochondrial mass (n=20–24) and ΔΨ_m_ (g-h), as determined by immunofluorescent staining (n=20–24) and JC-1 dye fluorescence in live cells (n=10–12). Also, 8-pCPT-cGMP reduces MPP+-induced loss of (i) mtDNA (n=19–22), (j) ATP (n=12) and (k) cell death quantified by Sytox green and DAPI fluorescence (n=22–24). Statistics were calculated using a one-way ANOVA with a post-hoc false discovery rate <0.05 (e-k).

To further determine the ability of cGMP signaling to maintain mitochondrial integrity and promote cell survival during rising levels of ROS, we next investigated the potential of cGMP signaling to protect against MPP+-induced mitochondrial toxicity. Preconditioning with 8-pCPT-cGMP protects MN9D neurons against MPP+-induced ROS accumulation (Fig. 6a–e) and loss of mitochondrial mass (Fig. 6f), ΔΨ_m_ (Fig. 6g–h), mitochondrial DNA (Fig. 6i), and ATP (Fig. 6j). Critically, while 8-pCPT-cGMP alone promotes MN9D neuron viability, preconditioning also rescues these neurons from MPP+-induced cell death (Fig. 6k).

## Discussion

Here, we reveal a protective role for GUCY2C against DA neuron degeneration. Functional GUCY2C is expressed by DA neurons in the nigrostriatal pathway, where it maintains mitochondrial content and function while limiting ROS accumulation. GUCY2C protects against neurodegeneration in MPTP mice, and cGMP, the mediator of GUCY2C signaling, plays a parallel role in reducing oxidative stress, protecting mitochondria from injury, and decreasing cell death in DA neurons in vitro. GUCY2C is overexpressed in DA neurons in the SNpc of patients with PD and mice treated with MPTP. These observations suggest that stimulating GUCY2C may be effective in preventing nigrostriatal neurodegeneration without off-target effects. Additionally, GUCY2C overexpression in pathology indicates the potential of GUCY2C as a biomarker of early PD pathology.

Our findings align with established data linking impaired mitochondria and increased ROS levels with PD development and susceptibility to neurodegeneration in mouse models of Parkinsonism.^27, 29–31, 72, 73^ Post-mortem analysis reveals that PD patients have elevated ROS in the nigrostriatal pathway and defects in ETC activity, mitochondrial biogenesis, mitophagy, and mitochondrial trafficking have been implicated in familial and idiopathic PD.^27–31^ *In vivo* experiments have determined that compensating for these deficiencies by upregulating antioxidant activity or restoring mitochondrial integrity by enhancing mitochondrial biogenesis, mitophagy, or even direct mitochondrial infusion rescues mice from MPTP pathology.^74–76^

Further studies are necessary to define the protective mechanisms of GUCY2C signaling in DA neurons. Although a specific role for GUCY2C in protecting against neurodegeneration is novel, cGMP signaling protects mitochondria, reduces ROS, and preserves survival across different cell types. In some cells, cGMP induces mitochondrial biogenesis by upregulating PGC1a,^77, 78^ a master transcription factor crucial to *de novo* mitochondria synthesis that is downregulated in SNpcs from *Gucy2c*^−/−^ mice. Empagliflozin, a sodium-glucose transport inhibitor used to treat diabetes, recently was demonstrated to play a separate role in reducing oxidative stress and cell death by cGMP signaling in a mouse model of diabetic cardiomyopathy.^79^ Moreover, cGMP upregulates antioxidant genes in colon, lung, and heart.^80–82^ Also, cGMP promotes homeostatic autophagy, a process dysregulated in PD,^83–86^ and at low levels, cGMP blocks apoptosis in cultured neuronal and neuroblastoma cells.^87, 88^ However promising these *in vitro* results may be for PD research, *in vivo* experiments investigating the role of cGMP in animal models of DA neurodegeneration have yielded conflicting results.^89, 90^ As previous experiments were performed using phosphodiesterase (PDE) inhibitors, which prevent degradation of cyclic nucleotides across a broad range of cells and pathways within the CNS, our study of a guanylyl cyclase selectively expressed by DA neurons represents the first potential *in vivo* approach to raise cGMP specifically within the nigrostriatal pathway.

To explain impaired mitochondria, elevated ROS levels, and susceptibility to neurodegeneration in *Gucy2c*^−/−^ mice, we have considered multiple potential mechanisms that may cause ROS overproduction or limit ROS scavenging in *Gucy2c*^−/−^ DA neurons. One possible cause of ROS overproduction begins with impaired mitochondrial dynamics. *Gucy2c*^−/−^ SNpcs express lower levels of PGC1a, a driver of mitochondrial biogenesis, and PINK1, a mediator of mitophagy and an indirect PGC1a activator that when mutated is associated with PD.^91–94^ Combined with lower levels of mitochondrial proteins in the nigrostriatal pathway, these observed deficiencies suggest that *Gucy2c*^−/−^ mice may have defective mitochondrial production and turnover, leaving DA neurons with a relatively limited and inefficient pool of mitochondria. As mitochondria are the primary source of cellular ROS,^95–97^ mitochondrial incompetence may have substantial consequences on cell integrity and resilience to insults. During homeostasis, oxidative phosphorylation generates multiple ROS species, including superoxide and downstream products like hydrogen peroxide (H_2_O_2_) and hydroxyl radical.^96^ At low levels, ROS are scavenged by antioxidants, including SOD2, catalase, and glutathione, but these defense mechanisms are overwhelmed when ROS production is significantly elevated in pathological conditions, such as when the ETC is impaired.^98, 99^ Reduced ETC complexes, oxygen consumption, and ATP production in the *Gucy2c*^−/−^ nigrostriatal pathway suggest impaired oxidative phosphorylation, which may result in overproduction of ROS.

Alternatively, the inciting source of potential ROS accumulation in *Gucy2c*^−/−^ DA neurons may precede mitochondrial dysfunction. In contrast to a previous study yielding no significant differences in striatal DA levels in *Gucy2c*^−/−^ mice,^100^ we detected a modest, yet significant, increase in DA in the *Gucy2c*^−/−^ striatum. DA-producing neurons are particularly vulnerable to oxidative stress in part because DA production and degradation yield substrates for autoxidation,^101^ which is typically offset by endogenous mechanisms. A recent study reveals that MAOs tethered to mitochondria in DA neurons redirect H_2_O_2_, a by-product of DA deamination to DOPAC, into complex IV of the ETC, thus limiting the ROS burden of DA metabolism while generating ATP.^102^ However, as *Gucy2c*^−/−^ mice produce more DA, but express lower levels of ETC proteins, they may simultaneously generate more ROS and lack the ability to reduce the resulting oxidative stress. Elevated oxidative stress induces DNA damage, peroxidation of lipid membrane, and protein oxidation, leading to a vicious cycle between ROS production and mitochondrial damage which may be amplified in *Gucy2c*^−/−^ mice.^103–105^

Another possible explanation of elevated oxidative stress and dysfunctional mitochondria is impaired ROS scavenging in *Gucy2c*^−/−^, which is supported by our *in vitro* findings. We and others have demonstrated that cGMP signaling promotes antioxidant function across a wide range of cell types, including cultured DA neurons.^80–82^ However, unlike our findings *in vivo*, our *in vitro* studies did not reveal a role for cGMP in promoting mitochondrial integrity in the absence of acute oxidative stress. This discrepancy may be due to the relatively short incubation time of cultured DA neurons with cGMP in contrast to the long-term impact of mice aging with competent GUCY2C expressed by SNpc DA neurons. Regardless, the precise role of GUCY2C in preventing ROS accumulation and mitochondrial dysfunction in DA neurons remains undefined and is a topic for future investigation. Comparing specific antioxidant enzyme activity between genotypes, treating *Gucy2c*^+/+^ and *Gucy2c*^−/−^ mice exposed to MPTP with antioxidants, and performing long-term *in vitro* characterizations of mitochondria within DA neurons supplemented with cGMP may help to determine the role of GUCY2C on ROS overproduction and scavenging.

The apparent feedback loop that exists between oxidative stress and GUCY2C expression adds another layer of complexity to the GUCY2C-mitochondria-ROS relationship. Notably, MPTP induces an upregulation of *Gucy2c* mRNA in the SNpc, but not in the VTA. As the VTA is comparitively resilient to mitochondrial toxins and has lower levels of oxidative stress at baseline,^57–64^ this observation supports the hypothesis that *Gucy2c* transcription is closely regulated by ROS sensors. GUCY2C is upregulated *in vivo* and *in vitro* in response to elevated ROS across species. Our data aligns with recent work exploring the relationship of ROS and cGMP in *C. elegans*. Mutating mitochondrial ETC complexes I or III or exposure to paraquat, a lipid membrane oxidation agent, increases cGMP accumulation that is prevented by pretreatment with antioxidants.^106^ Our study has identified *Gucy2c* as a potential transcriptional target of oxidative stress, which may hold translational implications for the utility of GUCY2C as a biomarker in PD. Currently, PD is diagnosed when patients lose 50–90% of the DA neurons within the SNpc and experience subsequent motor deficits.^107^ Although DAT single-photon emission computed tomography (DAT SPECT) is a highly effective FDA-approved technique to distnguish PD from other pathologies that induce essential tremor, there are currently no biomarkers that can identify early pathology prior to profound neurodegeneration.^5, 74^ Our data reveal GUCY2C as a ROS-responsive receptor expressed on 98% of TH+ SNpc neurons, and suggest that a GUCY2C ligand or antibody may have potential as a novel biomarker to identify early stages of neurodegeneration.

Many open questions remain for future investigation. Multiple epidemiological and experimental studies have implicated altered GI function and a disrupted gut-brain signaling axis in PD pathology. GI symptoms such as constipation and dysphagia frequently present in PD patients decades prior to motor deficits,^108, 109^ and alpha synuclein fibrils spread from the intestine to the brain via the vagus nerve, propogating Lewy body formation throughout the CNS.^110–114^ PD patients also have disrupted microbiomes, which may hold implications for neuroinflammation in pathology.^115–118^ Interestingly, the therapeutic potential of fecal transplants to ameliorate motor symptoms in PD patients is currently being explored in a clinical trial (ClinicalTrials.gov identifier NCT03808389). Disrupted endocrine signaling has been implicated in PD pathology. PD patients have lower levels of circulating ghrelin and GLP-1, GI hormones best known for their role in mediating satiety through hypothalamic signaling.^119, 120^ Recent experiments have revealed that ghrelin and GLP-1 have neuroprotective roles such as reducing neuroinflammation and mitigating DA neuron cell loss in MPTP mice.^121–126^ Importantly, we have previously demonstrated a gut-brain endocrine axis between uroguanylin, a hormone produced by the small intestine that can cross the blood-brain barrier, and hypothalamic GUCY2C in regulating satiety.^127, 128^ These findings suggest that intestinal uroguanylin may serve as the endogenous ligand for GUCY2C expressed in the nigrostriatal pathway. Studies comparing uroguanylin levels in PD and healthy patients, as well as ligand depletion and overexpression experiments in MPTP mice, are critical to determine whether nigrostriatal GUCY2C is a component of a novel neuroprotective gut-brain axis. Further, although loss of GUCY2C in mouse intestine did not increase vulnerability to MPTP, intestinal GUCY2C may play a larger role in alternative models of DA neuron degeneration. Determining the susceptibility of global vs conditional *Gucy2c*^−/−^ in other mouse models such as alpha-synuclein overexpression and *Pink1*^−/−^ may clarify neural and intestinal roles of GUCY2C in mitigating pathology.

## Materials and Methods:

### Mice

Mice for these studies were bred, maintained, genotyped, and functionally characterized in the animal care facility at Thomas Jefferson University. *Gucy2c*^−/−^ mice on a C57Bl6/J background are maintained within our colony.^129–133^ *Gucy2c^fl/fl^* mice were developed in conjunction with the CRISPR-Cas9 Mouse Targeting Core at University of Pennsylvania (Philadelphia, PA) (RRID:SCR_022378). *hTH-GFP* mice were generated as described and maintained on a C57Bl6/J background^.134^ *hTH-GFP* mice were crossed with *Gucy2c*^−/−^ mice to generate GUCY2C WT and KO reporter littermates. Villin^cre^ (Stock: 021504) were purchased from the Jackson Laboratory. Mice were raised with 12 h light and dark cycles and were used from age 12–24 weeks. All mice were compared to littermate controls or bred as F2 crosses of *Gucy2c*^+/+^ and *Gucy2c*^−/−^ from heterozygote parents.

### Brain immunofluorescence

Following perfusion of mice with ice-cold PBS followed by 4% paraformaldehyde, brains were extracted and incubated in 4% PFA at 4°C for 24–48 h, and then cryoprotected by incubation in 30% sucrose in PBS at 4°C until brains sank (24–72 h). Brains were frozen in OCT medium by submerging in dry ice-cooled methanol and stored at −80°C until sectioning. Forty-micron sections were cut using a cryostat, and then stored floating in cryoprotectant until staining. Each wash step below represents three consecutive 5-min incubations in PBS with 0.1% tween-20 (PBS-T). Epitope retrieval was performed on floating sections by incubating in pH 9 retrieval solution (Agilent Technologies, Santa Clara, CA) for 20 min at 80°C. Samples were blocked for 1 h in blocking buffer (10% milk (w/v) in PBS with 0.3% Triton-X), and then incubated, shaking in 1° antibody solution (diluted in blocking buffer) overnight at 4°C. After a wash step, samples were incubated, shaking in blocking buffer with 2° antibody and nuclear counterstain DAPI for 60 min at room temperature. For all stains except GUCY2C, a fluorophore-conjugated 2° antibody was used. To stain GUCY2C, a peroxidase-conjugated 2° antibody was used, followed by tyramine-amplification: samples were washed and then incubated in tyramide-FITC, at a final concentration of 100 μg/mL in PBS with 0.003% H2O2 for 10 min (Hopman et al. 1998). Following a final series of washes, samples were mounted onto Superfrost^™^ Plus Microscope Slides (Fisher Scientific, 12–550-15) slides with Prolong Diamond antifade mounting media (Thermo Fisher, Waltham, MA). Antibody IDs and concentrations are listed in Supplemental Table 1. Confocal images were taken of 10 different 40 μm sections from each mouse for each region. Cells were quantified from 5 different optical sections (at least 6 μm apart) for each 40 μM section. Mean fluorescent intensity was calculated in ImageJ software.

### RNAscope

Following perfusion of mice with ice-cold PBS, brains were extracted, frozen in OCT medium by submerging in dry ice-cooled methanol and stored at −80°C until sectioning. Ten-micron sections were cut using a cryostat onto Superfrost^™^ Plus Microscope Slides (Fisher Scientific, 12–550-15) and store at −80°C until use. RNAscope stained used the reagents and staining protocol provided by Advanced Cell Diagnostics (ACD) USA (Newark, CA) (ACD, 323110). Each wash step below represents two consecutive 2-min incubations in ACD wash buffer. Briefly, slides were drop-fixed in ice-cold 4% PFA for 15 min, followed by 5 min sequential dehydration steps in ice cold 50%, 70%, and 100% ethanol. Sections were blocked with 3% hydrogen peroxide for 10 min, followed by two wash steps and incubation with RNA probes (Supplemental table 1) for 2h at 40°C. Sections were then washed and incubated with AMP1 for 30 min, AMP2 for 30 min, and AMP3 for 15 min at 40°C with wash steps after each incubation. Depending on the RNAscope probe channel, sections were then incubated with C1, C2, or C3 for 15 min at 40°C, washed, and incubated with opal reagent 570 or 690 (Akoya Biosciences, Marlborough, MA) for 30 min at 40°C. Sections were washed and incubated in provided HRP blocking for 15 min at 40°C to complete the RNAscope stain. To combine RNAscope with immunofluorescence, sections were then blocked and counterstained with primary and secondary antibodies, following the immunofluorescence protocol detailed above (antibody concentrations for combined RNAscope and immunofluorescence provided in Supplemental table 1). DAPI was mixed into secondary antibodies to counterstain nuclei. Slides were coverslipped using Prolong Diamond antifade mounting media (Thermo Fisher). Confocal images were taken of 8 different 10 μm sections from each mouse for each region. Cells were quantified from 5 different optical sections (at least 6 μm apart) for each 10 μM section. Individual mRNA transcripts were analyzed in ImageJ software. The script for quantification is available upon request.

### Pretreatment of sections for 8-oxo-dG immunofluorescence

To detect 8-oxoG in mitochondrial DNA, free-floating sections were incubated in 10 mM Tris-HCl (pH 7.5), 15 mM NaCl containing DNase-free RNase (5 mg/ml of heat-inactivated RNase A, Sigma, Saint Louis, MO) for 60 min at 37°C, prior to incubation with primary antibody^.52^ As a negative control, cellular DNA was eliminated by incubating sections in 50 mM Tris-HCl (pH 7.5), 0.1 mM MgCl2 containing RNase-free DNase I (1000 U/ml, Sigma) for 60 min at 37°C, following incubation with DNase-free RNase. Antibody information is provided in Supplemental table 1.

### Cyclic GMP ELISA

Midbrains were isolated from *Gucy2c*^+/+^ and *Gucy2c*^−/−^ mice on ice and minced in Neurobasal media (Thermo Fisher) supplemented with 1X N-2, B-27, and GlutaMAX (Thermo Fisher). Minced midbrains were divided evenly into a 24w plate containing 150 uL of supplemented Neurobasal media with 3-isobutyl-1-methylxanthine (IBMX); 1 mM and incubated for 20 min at 37°C. One hundred and fifty uL of supplemented media containing 1mM IBMX and either 2 uM linaclotide (Ironwood Pharmaceuticals, Cambridge, MA) or 2 uM TJU (inactive peptide control) for an additional 30 min at 37°C. Subsequently, protein was purified (detailed below) and analyzed for cGMP using a competitive cGMP ELISA kit according to manufacturer instructions (Cayman Chemical Company, Ann Arbor, MI).

### Protein isolation

SNpc from *hTH-GFP* x *Gucy2c*^+/+^ and *hTH-GFP* x *Gucy2c*^−/−^ was isolated under a fluorescent microscope.^134^ Tissue was immediately placed into ice-cold M-PER (Thermo Fisher) supplemented with protease and phosphatase inhibitors (Roche) and homogenized using a 1 mL insulin syringe (BD biosciences). Striatal synaptosomes were isolated by homogenized dorsolateral striata in Syn-PER (Thermo Fisher) supplemented with protease and phosphatase inhibitors (Roche) and centrifugation according to manufacturer instructions. Protein was quantified using the BCA assay (Thermo Fisher).

### Immunoblot analysis

Lysates were analyzed by SDS-PAGE (NuPAGE 4-to-12% bis-Tris gel; Novex Life Technologies) and electrophoretically transferred to a nitrocellulose membrane (Novex Life Technologies). The membrane was blocked with 5% bovine serum albumin (BSA) in PBST (1× PBS and 1% Tween 20) and probed overnight at 4°C with primary antibodies detailed in Supplemental table 1. The following day, membranes were washed three times with PBST and incubated with goat anti-mouse horseradish peroxidase (HRP)-conjugated and goat anti-rabbit HRP-conjugated secondary antibodies (1:10,000; Jackson ImmunoResearch) in 5% BSA in PBST for 1 h at room temperature. Blots were developed in SuperSignal West Dura or Femto enhanced chemiluminescence (ECL) substrate (Thermo Scientific). Relative intensity was quantified by densitometry using ImageJ and normalized to the intensity of GAPDH or HSP90. Housekeeping probes were chosen to minimize interference with proteins of interest, after confirming that *Gucy2c*^−/−^ express comparable levels of HSP90 as *Gucy2c*^+/+^ (Supplemental fig. 2E–F).

### Synaptosome respirometry

For monitoring respiration, synaptosomes were resuspended in Seahorse medium (Agilent Technologies) (phenol-free DMEM pH 7.4, supplemented with 2 mM glutamine, 10 mM glucose, 1 mM pyruvate). Eighty μg synaptosomal protein per well was aliquoted into a 24 well of a cell culture microplate (Agilent Technologies) coated with a 1:15,000 diluted polyethyleneimine (PEI). The plate was centrifuged at 3,000g for 1 h at 4°C. The cell culture microplate was incubated and loaded into the Seahorse XF24 extracellular flux analyzer following the manufacturer’s nstructions. All experiments were performed at 37°C. Reagents were added at appropriate dilutions in seahorse medium (volumes and concentrations provided in Supplemental table 2). Three biological replicates and four technical replicates were run per plate.

### ATP assay

Synaptosomal and MN9D protein content was quantified prior to TCA deproteinization (Abcam, 204708). 10 uL of deproteinized sample was incubated with fluorescent ATP assay kit reagents (Abcam 83355) in a glass-bottom black 96w plate (Nalge Nunc International Corporation, Rochester, NY). Fluorescence ATP content was measured using a microplate reader at a 535/587 excitation/emission wavelength. Background levels of glycerol-3-phosphate were subtracted from each sample according to manufacturer instructions.

### Subtoxic MPTP

In experiments measuring GUCY2C levels following MPTP injections, *Gucy2c*^+/+^ mice received 4 intraperitoneal (IP) injections of vehicle or MPTP at 20 mg/kg (free base in PBS, Sigma). As this dose is toxic for *Gucy2c*^−/−^ mice (supplemental Fig. 6), experiments comparing *Gucy2c*^+/+^ vs Gucy2c^−/−^ response to MPTP used 4 IP injections of vehicle or MPTP at 10 mg/kg (referred to as subtoxic MPTP). Seven days after the last injection, all mice were heavily anesthetized with avertin prior to perfusion.

### TH Immunohistochemistry.

Every fifth 40 uM floating section of mouse midbrains was prepared and stained with primary antibody overnight as described above. Next, the sections were incubated with an avidin–biotin–horseradish peroxidase complex (Vector Laboratories) according to the manufacturer’s instructions. The sections were stained with a DAB kit (Vector Laboratories) before mounting onto Superfrost^™^ Plus Microscope Slides and coverslipping using Diamong ProLong mounting media.

### TH+ neuron counts

TH+ neurons were visualized using a 100×, 1.3 numerical aperture objective (Olympus, Center Valley, PA) on a BX51 microscope (Olympus) with a MAC5000 motorized XYZ axis computer-controlled stage and a CX9000 CCD video camera (MicroBrightField). Neurons were counted using a fractionator-sampling design in morphometry and design-based stereology software package, StereoInvestigator, (version 7.0; MicroBrightField, Colchester, VT, USA). Briefly, at least 8 sections of SNpcs were traced per mouse at 4x magnification, and this tracing was superimposed onto the 100x viewing field for neuron counting. The software randomly sampled predefined counting frames within each outline, allowing for an unbiased sampling of each SNpc section. Total neuron counts were estimated by factoring in the number of TH+ nuclei, size of SNpc section, and thickness of counting region. Further details on counting methods have previously been published.^135^

### HPLC analysis

Mouse brain issue was dissected, weighed, and homogenized in perchloric (0.3 N) acid for the HPLC/ED analysis. The HPLC system consisted of a solvent-delivery system (model 582 pump; ESA), an autosampler (model 542), and a coulometric electrochemical detector (Coulochem III; ESA). The guard cell was set to 350 mV. Electrodes 1 and 2 were adjusted to 150 mV and 250 mV, respectively. Chromatographic separations were performed on an MD-150, 3.2-m column, and the entire system was run at an ambient temperature. The mobile phase consisted of 85 mM sodium phosphate, 2 mM 1-octanesulfonic acid, 75 mM disodium EDTA, 0.02% triethylamine and 13% acetonitrile (vol/vol). The pH of the mobile phase was adjusted to 4.85 with sodium hydroxide. These solutions were prepared in HPLC-grade water, filtered through a 0.22-m membrane under vacuum, and pumped at a rate of 0.6 mL/min, producing a background pressure of 181 bars. Samples were further identified by spiking with external monoamine standards; the retention times (in min) of the monoamine standards and co-eluting peaks of samples were recorded. The concentrations of monoamines in the unknown samples were quantified by comparing the peak areas with those of the external monoamine standard chromatograms. All HPLC analysis was performed at Vanderbilt University.

### MAO-B enzyme activity

SNpc and striatal protein from *Gucy2c*^+/+^ and *Gucy2c*^−/−^ mice was homogenized in 200uL M-PER supplemented with protease inhibitor on ice. Ten uL of sample was immediately used in the Monoamine Oxidase Enzyme Activity kit (Sigma) in the presence of provided Clorgyline to inhibit MAO-A and isolate MAO-B activity. The protocol followed manufacturer instructions. Results were normalized to individual sample protein content, as determined by the BCA assay.

### Messenger RNA isolation, DNA isolation, and qRT-PCR

Messenger RNA isolation was performed on tissue using the RNeasy Plus Mini kit (Qiagen, 74034) and stored at −80°C for further processing. Reverse transcription was performed with TaqMan Reverse Transcription Reagents (Thermo Fisher, N8080234) according to manufacturer’s instructions. DNA isolation was performed using the QIAamp DNA Micro (Qiagen 56304). Quantitative RT-PCR was performed using Applied Biosystems TaqMan Master Mix (Thermo Fisher) for cDNA, or with PowerUP Sybr Master Mix (Thermo Fisher) for DNA. Primer probes are listed in Supplemental Table 2. Relative expression was calculated as 2^−ΔΔCt^.

### MN9D culture conditions

MN9D neurons were generously provided by Dr. Zigmond (University of Pittsburgh) and were cultured according to modified developer instructions.^66^ Briefly, MN9D cells were plated on 1% poly-L-lysine (Sigma) in DMEM/F-12 media (Thermo Fisher) supplemented with 10% FBS and 1% penicillin/streptomycin at a density of 30K/cm^2^. MN9Ds cultured in serum-starved media were grown in identical conditions, DMEM/F12 supplemented with 5% FBS for the final 24 h in culture. To differentiate MN9Ds, media was supplemented with 1 mM butyric acid for 4 d.^136^ All experiments were performed on differentiated neurons. Cell permeable 8-pCPT-cGMP (Biolog Life Science Institute, Germany) was used at a concentration of 100 uM for 48 h.^137–139^ MPP+ was used at 100 uM for 24 h.^58, 140–143^

### MN9D experiments

Across every experiment, each data point shown represents the average of one well. ATP activity was determined as described above. DNA was isolated as described above. MtDNA was determined by normalizing mtDNA-encoded 16S ribosomal RNA to nuclear DNA-encoded beta-2 microglobulin. As MPP+ reduced mtDNA to undetectable levels in a substantial number of MN9D samples, mtDNA is presented as a binary positive or negative value with a threshold of 20% of average baseline expression. Antioxidant capacity of MN9D lysates was determined using the total antioxidant capacity assay kit (Abcam) according to manufacturer instructions. To measure ROS via CM-H2DCFDA (Thermo Fisher), MN9Ds were cultured and treated in black, glass-bottomed 96w plates in phenol-free media and incubated with CM-H2DCFDA according to manufacturer instructions. Fluorescent signal was quantified in a plate reader at a 480/520 excitation/emission wavelength. Cells were next fixed in 4% PFA with DAPI for 10 m at 4° and washed once with PBS. Fluorescent signal was quantified via plate reader at a 355/460 excitation/emission wavelength. Each sample is presented as CM-H2DCFDA signal normalized to DAPI signal. To determine cell viability by fluorescent alamarBlue^™^ assay, MN9Ds were cultured and treated in black, glass-bottomed 96w plates (Nalge Nunc International Corporation) in phenol-free media and incubated with alamarBlue^™^ (Thermo Fisher) according to manufacturer instructions. To measure ΔΨm using JC-1 dye, MN9Ds were cultured on black 96 well glass-bottomed well plates and incubated with JC-1 (Thermo Fisher) according to manufacturer instructions. Fluorescent signal of live cells was quantified in a plate reader at a 480/520 and a 584/612 excitation/emission wavelength. To determine ΔΨm using MitoTracker^™^, mitochondrial distribution, and ROS levels, MN9Ds were cultured on glass coverslips and co-incubated with MitoTracker^™^ Red CMX Ros (Thermo Fisher) and CellROX^™^ Far Red (Thermo Fisher) according to manufacturer instructions. Stained and fixed neurons were imaged on a confocal microscope. Mitochondrial membrane potential was measured by calculating mean fluorescent intensity of MitoTracker^™^ within neuronal cytoplasm using ImageJ software. Mitochondrial distribution was calculated by converting positive MitoTracker^™^ signal into a binary mask using ImageJ software. Percent area of cytoplasm that has a positive MitoTracker^™^ signal is shown in Figure 6f. The script for quantification is available upon request. To measure ROS using CellROX^™^, the mean fluorescence intensity of CellROX^™^ was measured within neuronal cytoplasm. Cell death was determined by plating and treating MN9D neurons in a black, glass-bottomed 96w plate (Nalge Nunc International Corporation) in phenol-free media. Live cells were incubated with cell death marker Sytox Green (Thermo Fisher) according to manufacturer instructions. Fluorescent signal was quantified by a plate reader at a 480/520 excitation/emission wavelength. Cells were then washed with PBS and fixed in 4% PFA and DAPI at 4°C for 10 min. Fluorescent signal of DAPI was quantified by a plate reader at a 390/410 excitation/emission wavelength.

### Dataset Acquisition and Analysis

Human midbrain microarray data were downloaded from Gene Expression Omnibus ID [dataset] GEO: GSE42966 on February 17, 2021. GSE42966 was based on the Agilent GPL4133 platform (Agilent-014850 Whole Human Genome Microarray 4×44K G4112F). Data were freely available online, and our study did not involve any experiments with humans or animals performed by any of the authors. The GEO2R online analysis tool (https://www.ncbi.nlm.nih.gov/geo/geo2r/) was used to detect gene levels of Th, Gucy2c, and Vmat2 (supplemental Fig. 4A).

### Statistics

Results are presented as the mean ± SEM, and a p value of <0.05 was considered significant. Statistical analysis was performed in Graphpad Prism 9 (Version 9.3.1) unless otherwise stated. In Figure 1l, RNAscope values were analyzed using a one-tailed t-test. In Figure 1n, cGMP values were analyzed using a two-way ANOVA with a post-hoc false discovery rate <.05. In Figures 2c, f, i, j, k, n, and q, values were analyzed using a one-tailed t-test. In Figures 2g and h, statistics were calculated using a two-way ANOVA with a post-hoc false discovery rate <.05. Figure 3 statistics were calculated using a two-way ANOVA with a post-hoc false discovery rate <.05. In Figure 4a, b, l, and j, statistics were calculated using a one-tailed t-test. In Figures 4c and d, statistics were calculated using a two-way ANOVA with a post-hoc false discovery rate <.05. In Figures 4e and f, statistics were calculated using a one-way ANOVA with a post-hoc false discovery rate <.05. In Figure 5b–e, statistics were calculated using a one-tailed t-test. In Figure 5f, statistics were calculated using a two-way ANOVA with a post-hoc false discovery rate <.05. In Figures 6e–k, statistics were calculated using a one-way ANOVA with a post-hoc false discovery rate <.05.

### Study Approval

The Thomas Jefferson University Institutional Animal Care and Use Committee approved all animal protocols and procedures under protocols 01357 and 01892.

## Supplementary Material

Supplement 1

## Abbreviations:

SNpc: substantia nigra pars compacta
PD: Parkinson’s disease
cGMP: cyclic guanosine monophosphate
ROS: reactive oxygen species
MPTP: 1-methyl-4-phenyl-1 2 3 6-tetrahydropyridine
GUCY2C: guanylyl cyclase C
DA: dopamine
ETC: electron transport chain
PDE: phosphodiesterase
L-DOPA: levodopa
MAO: monoamine oxidase
DOPAC: 3,4-dihydroxyphenylacetic acid
HVA: homovanillic acid
GLP-1: glucagon-like peptide 1
