# Supplementary material for: GUCY2C signaling limits dopaminergic neuron vulnerability to toxic insults
Source: Res Sq. 2023 Oct 13:rs.3.rs-3416338. Preprint. [Version 1] doi: 10.21203/rs.3.rs-3416338/v1 (PMC10602097; doi:10.21203/rs.3.rs-3416338/v1)
Supplement: Supplement 1 [file NIHPPrs3416338v1-supplement-1.pdf]

Supplemental Figure 1. Stimulating SNpc GUCY2C does not induce extracellular release of cGMP.

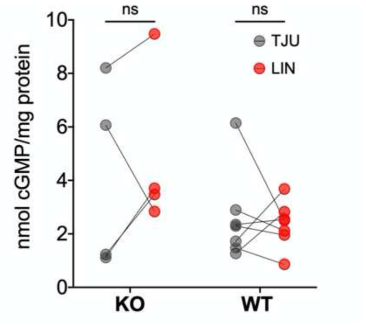

Linaclotide stimulation does not induce extracellular accumulation of cGMP in the *Gucy2*$^{+/+}$ or *Gucy2c*$^{-/-}$ SNpc, as determined via cGMP ELISA analysis of supernatant (n=4-7). Statistics were calculated using a two-way ANOVA with a post-hoc false discovery rate <0.05.

Supplemental Figure 2. GUCY2C supports mitochondrial electron transport complexes within the nigrostriatal pathway.

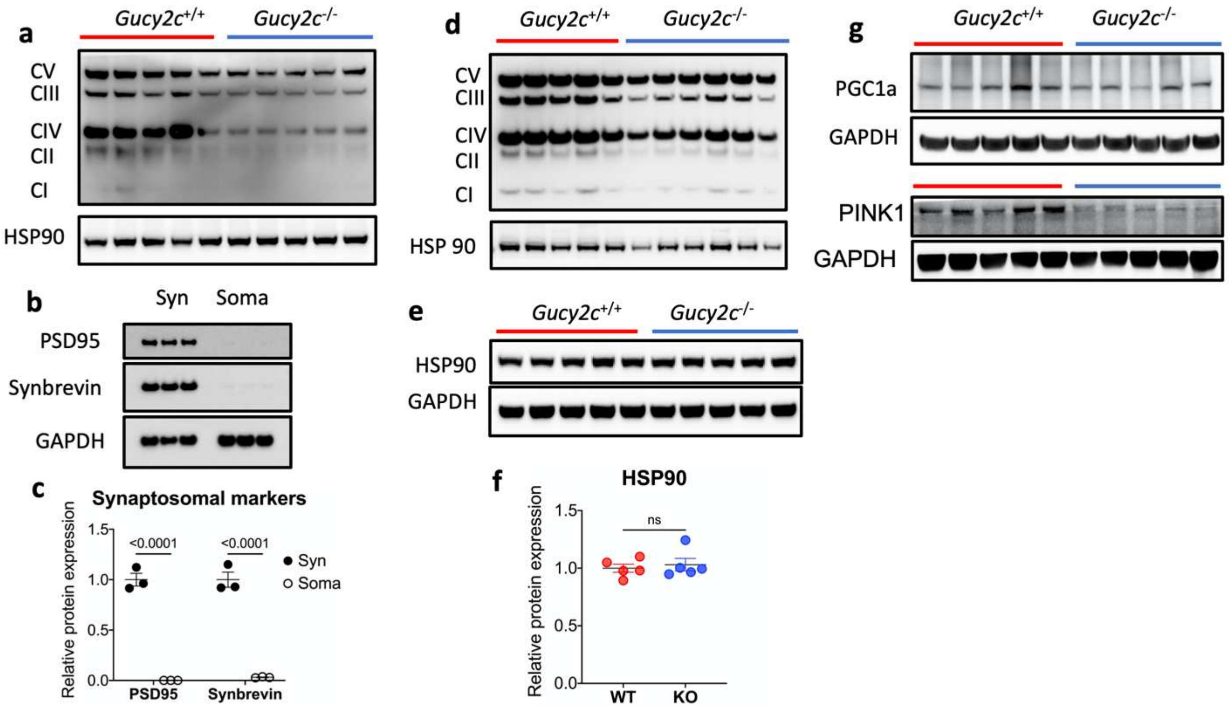

(a-d) *Gucy2c*$^{-/-}$ mice express significantly lower levels of mitochondrial ETC proteins in the **(a)** SNpc and **(b-d)** striatal synaptosomes compared to *Gucy2c*$^{+/+}$ mice (n=5-6). To avoid interference with ETC protein bands, this data is normalized to HSP90, which is **(e-f)** expressed at comparable levels in *Gucy2c*$^{+/+}$ and *Gucy2c*$^{-/-}$ SNpc's (n=5). **(g)** *Gucy2c*$^{-/-}$ mice express significantly lower levels of PGC1a and PINK1 protein in the SNpc as compared to *Gucy2c*$^{+/+}$ mice (n=5). Statistics were calculated using two-way ANOVA with a post-hoc false discovery rate <0.05 (c) and a one-tailed t-test (f).

Supplemental Figure 3. GUCY2C and dopaminergic neuron vulnerability

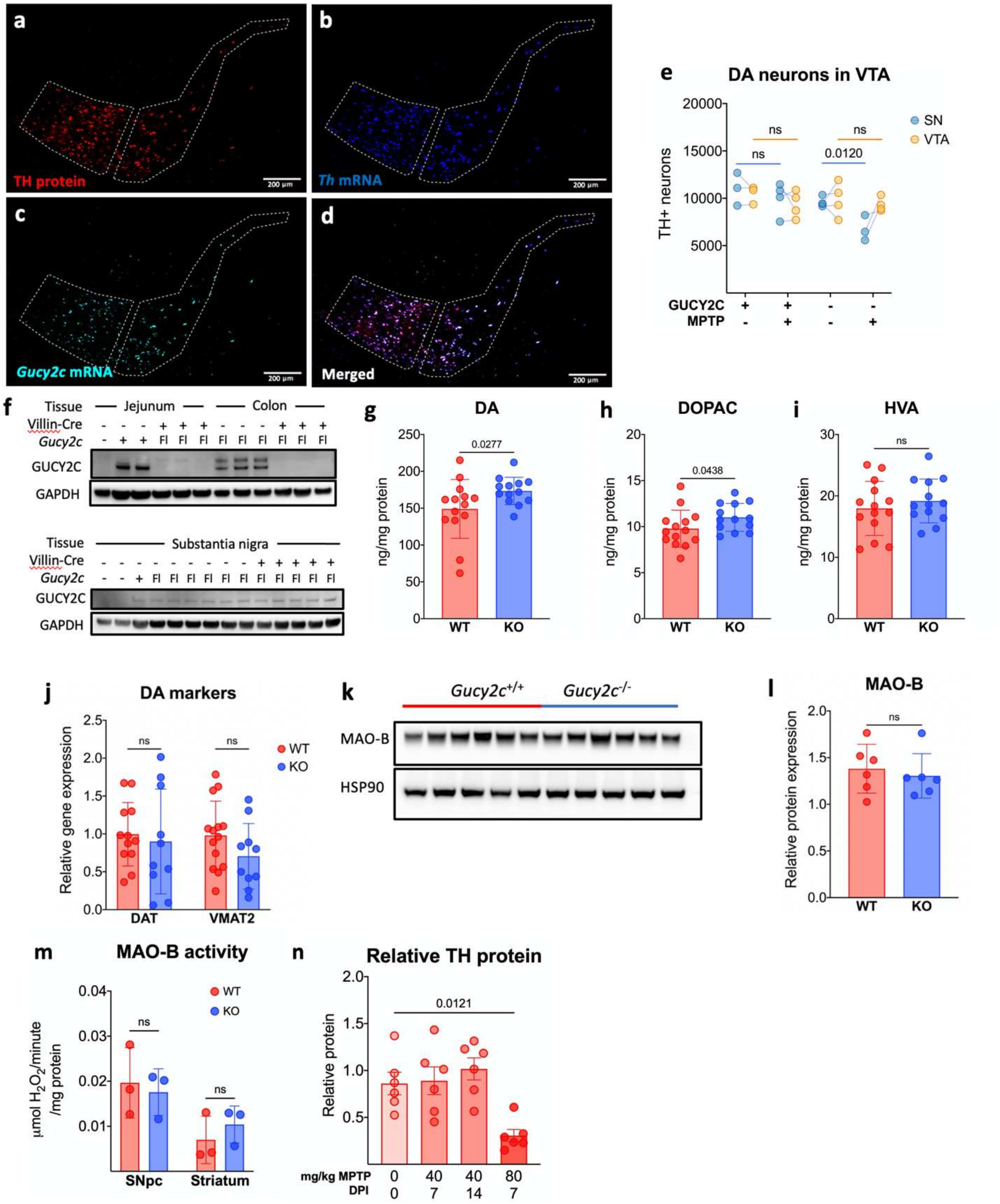

(a-e) *Gucy2c*$^{+/+}$ and *Gucy2c*$^{-/-}$ mice do not lose TH+ neurons in the VTA following a subtoxic dose of MPTP (n=3-4). **(f)** The Villin^{cre} x *gucy2c*$^{fl/fl}$ mouse selectively loses GUCY2C from the intestine, but not from the SNpc (n=3-5); [(-), *Gucy2c*$^{-/-}$; (+), *Gucy2c*$^{+/+}$; (Fl), *Gucy2c*$^{fl/fl}$]. **(g-i)** *Gucy2c*$^{-/-}$ mice

have higher levels of DA and DOPAC, but not HVA, at baseline as compared to *Gucy2c*$^{+/+}$ mice (n=13-14). **(j)** *Gucy2c*$^{+/+}$ and *Gucy2c*$^{-/-}$ mice express comparable levels of DAT and VMAT2 mRNA within TH+ DA neurons (n=10-14). **(k-l)** *Gucy2c*$^{+/+}$ and *Gucy2c*$^{-/-}$ mice express comparable levels of MAO-B protein in the SNpc (n=6) and have **(m)** comparable activity levels of MAO-B in the nigrostriatal pathway (n=3). **(n)** Unlike 80 mg/kg of MPTP, subtoxic MPTP does not induce a loss of TH protein in the *Gucy2c*$^{+/+}$ SNpc at one or two weeks post injection (n=6). Statistics were calculated using two-way ANOVA with a post-hoc false discovery rate <0.05 (e, j, m, n) and a one-tailed t-test (g-i, l).

Supplemental Figure 4. GUCY2C is overexpressed in pathology.

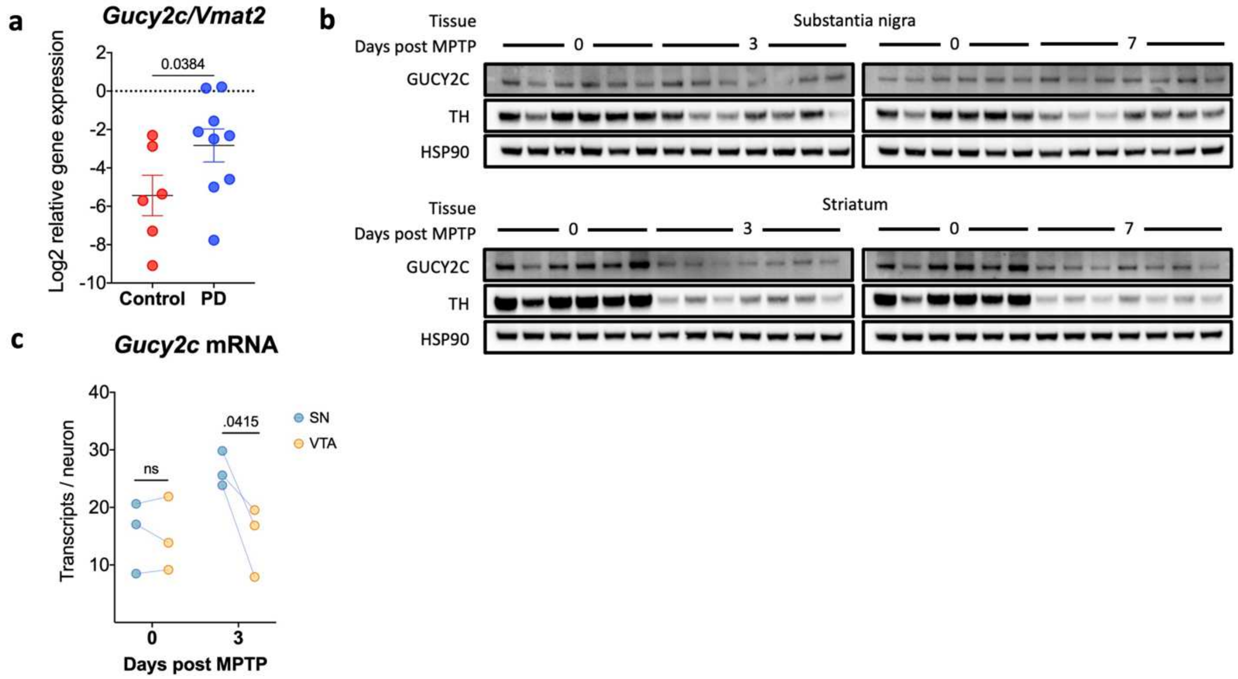

(a) *Gucy2c/Vmat2* mRNA is higher in PD patients as compared to control (n=6-9). **(b)** GUCY2C protein is increased relative to TH in the nigrostriatal pathway following MPTP (n=5-7). **(c)** *Gucy2c* mRNA is upregulated in the *Gucy2c*$^{+/+}$ SNpc, but not in the VTA, post-MPTP (n=3). Statistics were calculated using a one-tailed t-test (a) and two-way ANOVA with a post-hoc false discovery rate <0.05 (b-c).

Supplemental Figure 5. MN9D neurons are responsive to cGMP signaling.

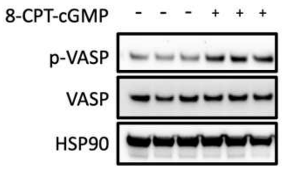

8-pCPT-cGMP increases the phosphorylation of VASP in MN9D neurons (n=3). Statistics were calculated using a one-tailed t-test.

Supplemental Figure 6. Standard MPTP dosing regiment induces lethal toxicity in *Gucy2c*$^{-/-}$ mice.

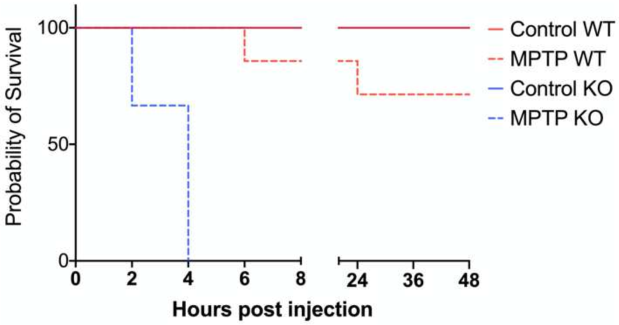

IP injections of 20 mg/kg of MPTP (standard dosing regimen for acute model) induces lethal toxicty in 100% of *Gucy2c*$^{-/-}$ mice (n=3-10).

Supplemental table 1. Antibody and RNAscope probe information

Antibody	Application	Host	Concentration	Company	Catalog number
TH	IF	Chicken	1:500	Abcam	ab76442
TH	IF, IHC	Rabbit	1:1000	Pel-Freez Biologicals	p40101
Iba1	IF	Rabbit	1:1000	Wako	019-19741
GFAP	IF	Chicken	1:1000	Millipore	AB5541
GUCY2C	IF, IB	Mouse IgG2a	1ug/mL	In-house	N/A
TMEM119	IF	Rabbit	1:1000	Cell Signaling Technology	83308
VDAC1	IF	Rabbit	1:1000	Thermo Fisher	PA1-954A
TOM20	IF	Rabbit	1:1000	Thermo Fisher	MA5-32148
8-oxo-dG	IF	Mouse IgG1	1:100	Abcam	ab145595
beta-III-tubulin	IF	Rabbit	1:1000	Abcam	ab18207
Total OXPHOS Rodent WB cocktail	IB	Mouse IgG1	1:250	Abcam	ab110413
PGC1a	IB	Rabbit	1:1000	Novus	NBP1-04676SS
PINK1	IB	Rabbit	1:1000	Thermo Fisher	PA1-4515
pVASP ser239	IB	Rabbit	1:1000	Cell Signaling Technology	3114
VASP	IB	Rabbit	1:1000	Cell Signaling Technology	3132

Probe	Application	Channel	Concentration	Company	Catalog number
GUCY2C	RNAscope	C1	As supplied	ACD	436591
TH	RNAscope	C2	1:50	ACD	317621-C2
PINK1	RNAscope	C3	1:50	ACD	524081-C3
Opal 520	RNAscope	520	1:1000	Akoya	FP1487001KT
Opal 690	RNAscope	690	1:1000	Akoya	FP1497001KT

IF = immunofluorescence, IHC = immunohistochemistry, IB = immunoblot

Supplemental table 2. qPCR primer probe information

Probe	Sequence/ Catalog number	Company	Reagents
msGUCY2C	Mm01267705_m1	ThermoFisher	Taqman
msTH	Mm00447557_m1	ThermoFisher	Taqman
ms16SR FWD	CCGCAAGGGAAAGATGAAAGAC	EuroFins	PowerUP Syber Green
ms16SR REV	TCGTTTGGTTTCGGGGTTTC	EuroFins	PowerUP Syber Green
msB2M FWD	ATGGGAAGCCGAACATACTG	EuroFins	PowerUP Syber Green
msB2M REV	CAGTCTCAGTGGGGGTGAAT	EuroFins	PowerUP Syber Green

ms = mouse, 16SR = 16S ribosomal RNA (mitochondrial-encoded gene), B2M = beta-2-microglobulin (nuclear-encoded gene)
